# Azacitidine Beyond the Bone Marrow: An Unexpected Journey Into Azacitidine-Induced Arthropathy

**DOI:** 10.7759/cureus.97020

**Published:** 2025-11-16

**Authors:** Syamasis Bandyopadhyay, Arghya Sahu, Sandip Kumar Chandra, Aheli Ghosh Dastidar, Sourav Pratihar

**Affiliations:** 1 Internal Medicine, Apollo Hospitals, Kolkata, IND

**Keywords:** acute myeloid leukemia, azacitidine, drug-induced arthropathy, hypomethylating agents, naranjo score, seronegative arthritis

## Abstract

Azacitidine, a DNA hypomethylating agent, is a cornerstone in the treatment of myelodysplastic syndromes and acute myeloid leukemia (AML) in patients unfit for intensive chemotherapy. While its adverse effect profile is well-documented, musculoskeletal complications such as inflammatory arthropathy are exceedingly rare and underrecognized. We report a 61-year-old male with AML, chronic kidney disease, and hypertension, who developed seronegative inflammatory arthropathy during the sixth and seventh cycles of intravenous chemotherapy. The patient presented with bilateral shoulder joint pain, marked morning stiffness, and a restricted range of motion, particularly affecting the left side. Inflammatory markers were elevated, while autoimmune serologies, including rheumatoid factor (RF), anti-cyclic citrullinated peptide antibodies (anti-CCP), antinuclear antibodies (ANA), and human leukocyte antigen B27 (HLA-B27), were negative. The clinical and temporal profile, along with symptom resolution following intra-articular corticosteroid administration and gabapentin therapy, supported a diagnosis of azacitidine-induced arthropathy. The Naranjo Adverse Drug Reaction Probability Score was 9, indicating a definite causal relationship. This case underscores a rare but important immune-mediated adverse effect of azacitidine: seronegative inflammatory arthropathy. As the use of hypomethylating agents expands, awareness of such atypical complications is critical. Early recognition can prevent diagnostic delays, reduce unnecessary investigations, and allow appropriate therapeutic intervention without compromising oncologic care. Further research is needed to elucidate underlying mechanisms and identify at-risk populations.

## Introduction

Azacitidine, a pyrimidine nucleoside analog of cytidine, is an epigenetic modulator and DNA methyltransferase inhibitor that has become a key therapeutic option in the management of myelodysplastic syndromes (MDS) and acute myeloid leukemia (AML), particularly in patients unfit for intensive induction chemotherapy or stem cell transplantation [1]. Through incorporation into both DNA and RNA, azacitidine inhibits DNA methyltransferase activity, leading to global DNA hypomethylation and reactivation of silenced tumor suppressor genes, while interference with RNA metabolism contributes to cytotoxicity at higher doses [2]. These dual actions account for its therapeutic efficacy but also underlie a spectrum of adverse effects.

The toxicity profile of azacitidine is well characterized, with common adverse events including cytopenias, gastrointestinal intolerance, and injection-site reactions [3]. However, musculoskeletal adverse effects, particularly inflammatory arthropathy or arthritis, are exceptionally uncommon, underrecognized, and sparsely documented in the literature. Only isolated case reports have described azacitidine-associated inflammatory or reactive arthritis in patients with hematological malignancies, suggesting a possible immune-mediated mechanism. Given that such manifestations can mimic autoimmune or paraneoplastic arthritis, recognition of drug-induced causes is often delayed, leading to unnecessary investigations and treatment interruptions.

The rarity and poorly understood pathogenesis of azacitidine-induced inflammatory arthropathy make it a clinically significant adverse event worth reporting. Awareness of this potential complication is crucial for oncologists, rheumatologists, and internists, especially as the use of hypomethylating agents expands beyond MDS and AML into other hematologic and even solid malignancies. We herein present a case of azacitidine-induced seronegative inflammatory arthropathy in a patient with AML, emphasizing its clinical presentation, diagnostic considerations, and therapeutic response, while discussing possible underlying mechanisms and literature correlations.

## Case presentation

**Figure 1:**
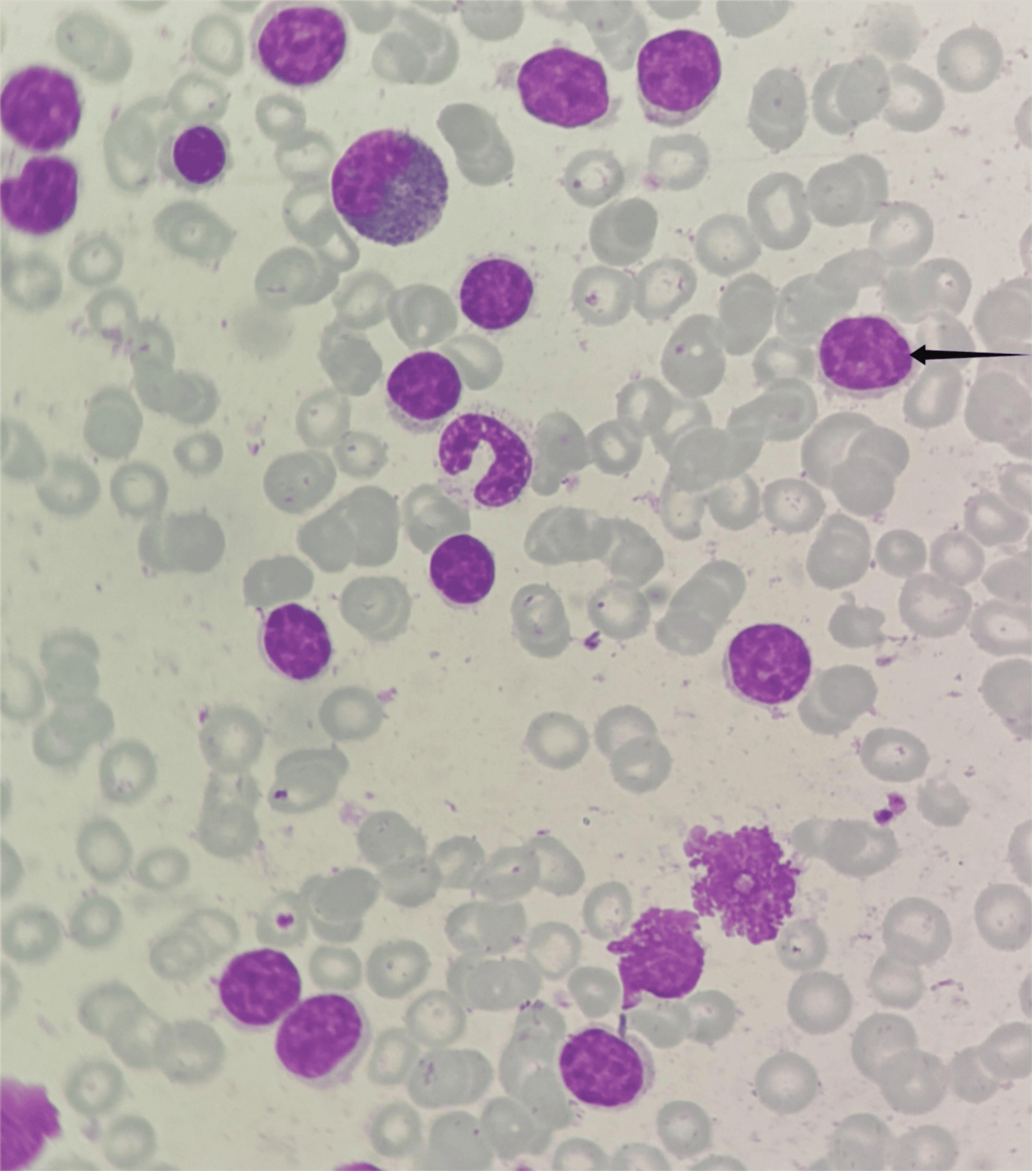
Bone marrow aspiration cytology showing blast cell (arrow) with a high nuclear-to-cytoplasmic ratio and fine chromatin, suggestive of acute myeloid leukemia.

**Figure 2:**
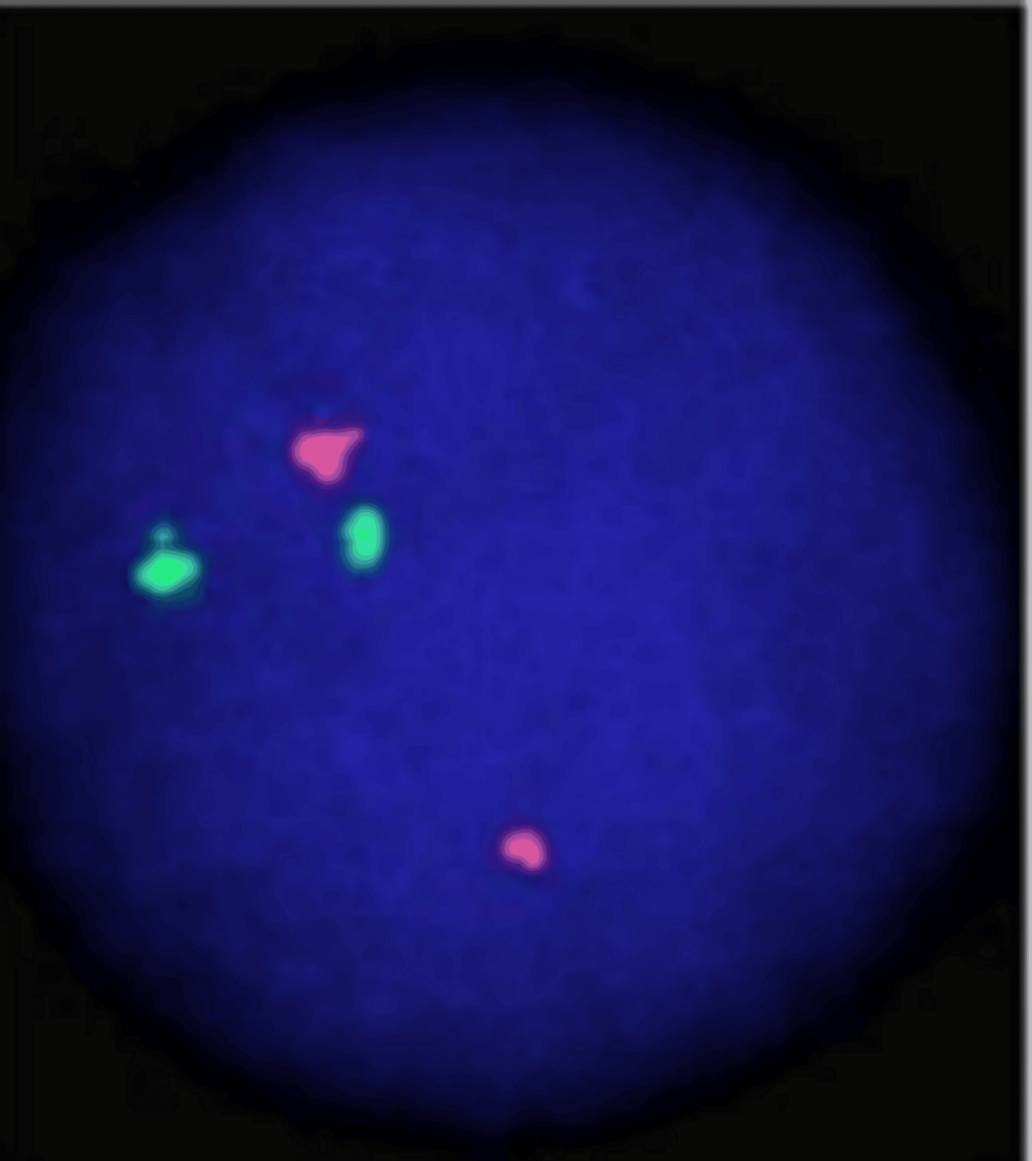
Fluorescence in situ hybridization (FISH) showed a 2R2G pattern without fusion signals, indicating a promyelocytic leukemia-retinoic acid receptor alpha (PML-RARA)-negative result.

A 61-year-old man with no prior history of autoimmune disease, gout, or joint complaints was diagnosed with acute myeloid leukemia after presenting with gradually progressive dyspnea and persistent anemia. Bone marrow aspiration, cytology, and biopsy revealed acute leukemia with 63% blast cells (Figure 1). Flow cytometry was suggestive of acute myeloid leukemia, and the fluorescence in situ hybridization (FISH) panel for promyelocytic leukemia-retinoic acid receptor alpha (PML-RARA) was negative (Figure 2). The patient was a known case of chronic kidney disease (G3a/A3) and hypertension. The patient was started on intravenous azacitidine (the dose was 100 mg intravenous infusion for the first 5 days, then 200 mg intravenous infusion for the remaining two days) and tablet Venetoclax (100 mg) for seven consecutive days every 28 days. On admission for the fourth cycle of chemotherapy, the patient developed prolonged neutropenia requiring granulocyte macrophage colony-stimulating factor injection (GM-CSF); therefore, the duration of intravenous azacitadine was reduced to five days per cycle. (Only 100 mg intravenous infusion for five days was given.) His baseline creatinine was 1.5 mg/dL, and creatinine clearance was 46 mL/minute. Therefore, no renal dose adjustment was made (creatinine clearance was > 30 mL/min). During therapy and post-therapy, his creatinine level and urine output were closely monitored. Even after five cycles of chemotherapy, there was no reduction in urine output or rise in serum creatinine level. On admission for the sixth cycle and seventh cycle of chemotherapy, the patient developed bilateral shoulder joint pain with significant morning stiffness without any associated fever, rash, or conjunctivitis. Symptoms were progressive, asymmetric (left> right), and inflammatory in character. The patient was afebrile and hemodynamically stable at presentation. Musculoskeletal examination revealed pain and stiffness in the shoulder with progressive limitation of active and passive range of movements across all planes, particularly external rotation and abduction. Tenderness on palpation was noted around the shoulder joint, more pronounced on the left side. Laboratory investigations revealed elevated C-reactive protein (CRP) and antistreptolysin O (ASO) titers, while the uric acid level was normal. In view of suspected inflammatory arthropathy, relevant investigations were conducted, which revealed negative rheumatoid factor (RF), anti-cyclic citrullinated peptide (anti-CCP) antibodies, antinuclear antibodies (ANA), and human leukocyte antigen B27 (HLA-B27) (Table 1). The patient was managed with intra-articular infiltration of triamcinolone (40 mg) in the left shoulder, along with oral gabapentin therapy, which was gradually up-titrated from 100 mg daily to 450 mg daily, and continued for two months. Supportive measures included local ice application and shoulder mobilization exercises. This regimen resulted in significant improvement in bilateral shoulder joint pain.

**Table 1:** Relevant laboratory investigations supporting azacitidine-induced inflammatory arthropathy, ruling out other causes. CRP, C-reactive protein; ASO, antistreptolysin O; ANA, antinuclear antibody; anti-CCP, anti-cyclic citrullinated peptide; RA, rheumatoid factor; HLA-B27, human leukocyte antigen B27

Laboratory test	Result	Interpretation/Reference range
CRP	7.3 mg/dL (elevated)	Normal: <0.5 mg/dL
ASO titer	1120 (elevated)	Normal: <200 IU/mL
ANA	Negative	Normal: Negative
Anti-CCP	Negative	Normal: Negative
RA factor	Negative	Normal: Negative
HLA-B27	Negative	Normal: Negative
Uric acid	5.6 mg/dL	Normal: 2.6-7.2 mg/dL

## Discussion

The pathophysiology of azacitidine-induced arthropathy remains incompletely understood but is likely multifactorial, involving immune dysregulation and epigenetic reprogramming. Azacitidine induces global DNA hypomethylation and transcriptional reactivation of previously silenced genes, which, in turn, enhances expression of endogenous retroelements and cancer-testis antigens, resulting in *viral-mimicry*-like activation of innate immune pathways through interferon and pattern-recognition receptor signaling [4]. The ensuing activation of type I interferon, tumor necrosis factor-α (TNF-α), and interleukin-6 (IL-6) pathways creates a systemic pro-inflammatory milieu capable of precipitating immune-mediated arthropathy in predisposed individuals [4]. Azacitidine also modulates T-cell differentiation and cytokine signaling, particularly downregulating IL-6/STAT3 and FOXP3-negative CD4⁺ effector cells, which can disturb immune homeostasis and trigger autoimmune inflammation in non-target tissues, including joints [5].

Clinical reports describe recurrent or reactive arthritis temporally associated with azacitidine therapy, typically occurring within days after drug administration and resolving upon drug withdrawal or corticosteroid therapy, supporting an immune-mediated hypersensitivity mechanism rather than direct cartilage toxicity [1,6]. In patients with hematological malignancies, the differential diagnosis of inflammatory arthritis is broad and includes paraneoplastic arthritis, which typically presents around the time of cancer diagnosis, persists unless the underlying malignancy is treated, and is usually unresponsive to intra-articular corticosteroid infiltration or gabapentin; autoimmune arthritis, which generally has a longer prodromal phase, systemic manifestations, and rarely presents abruptly or resolves completely within days; and drug-induced arthritis, which is often overlooked. Its diagnosis relies on temporal correlation with drug exposure and exclusion of other possible causes [6].

In this patient, arthritis developed during the sixth cycle of chemotherapy and worsened during the seventh. It was inflammatory in nature, seronegative, and responded adequately to intra-articular triamcinolone infiltration and gabapentin. The absence of systemic features, autoantibodies, and infectious etiology, along with the resolution of symptoms following drug discontinuation and steroid administration, supports a diagnosis of azacitidine-induced seronegative inflammatory arthropathy.

The Naranjo Adverse Drug Reaction Probability Scale is a validated method for determining the likelihood of a drug related adverse event [7]. This patient yielded a total score of 9 (2 + 1 + 1 + 2 + 2 + 1 = 9), indicating a definite causal relationship between azacitidine administration and the development of inflammatory arthropathy.

Key elements contributing to the score includes appearance of adverse event after administration of the suspected drug (score 2), presence of objective evidences (score 1), symptom resolution with drug withdrawal (score 1), reappearance of the adverse reaction on re-administration of the drug (score 2), exclusion of alternative diagnosis (score 2), and known association in literature (score 1).

## Conclusions

This case underscores a rare but important adverse effect of azacitidine, seronegative inflammatory arthropathy, occurring in a patient with acute myeloid leukemia during intravenous therapy. The clinical presentation may mimic autoimmune or paraneoplastic arthritis, especially in patients with hematological malignancies such as acute myeloid leukemia. As the use of hypomethylating agents continues to expand in hematological oncology, clinicians must remain vigilant for atypical immune-mediated side effects, including inflammatory arthropathy. Because it is a rare condition, clinicians should not overlook azacitidine as a possible cause of arthritis exacerbation when arthritis of unknown etiology develops in patients treated with azacitidine.

This case highlights the importance of maintaining a high index of suspicion for drug-induced etiologies when patients present with new-onset seronegative arthropathy during azacitidine therapy. The temporal relationship with drug administration, rapid resolution with corticosteroids, and absence of any other causative factors support the diagnosis. Early recognition and appropriate management can prevent unnecessary investigations and allow safe modification of therapy without compromising disease control. Further research is warranted to clarify the immunopathogenic mechanisms and to better define risk factors for such adverse events.

## References

[1] Alsaadi D, Low L, Zubairu M, Clesham K, Rowan F (2023). Azacitidine as a rare cause of reactive arthritis in a patient with acute myeloid leukemia. J Oncol Pharm Pract.

[2] Khan C, Pathe N, Fazal S, Lister J, Rossetti JM (2012). Azacitidine in the management of patients with myelodysplastic syndromes. Ther Adv Hematol.

[3] Wei AH, Döhner H, Pocock C (2020). Oral azacitidine maintenance therapy for acute myeloid leukemia in first remission. N Engl J Med.

[4] Johnson AJ, Yeh YY, Smith LL (2012). The novel cyclin-dependent kinase inhibitor dinaciclib (SCH727965) promotes apoptosis and abrogates microenvironmental cytokine protection in chronic lymphocytic leukemia cells. Leukemia.

[5] Lamprianidou E, Kordella C, Kazachenka A (2021). Modulation of IL-6/STAT3 signaling axis in CD4+FOXP3- T cells represents a potential antitumor mechanism of azacitidine. Blood Adv.

[6] Iltar U, Alhan FN, Vural E (2022). Recurrent arthritis as an unexpected side effect associated with azacitidine in a patient with myelodysplastic syndrome. J Oncol Pharm Pract.

[7] Busto U, Naranjo CA, Sellers EM (1982). Comparison of two recently published algorithms for assessing the probability of adverse drug reactions. Br J Clin Pharmacol.

